# Different serum sodium assay, different model for end stage liver disease - sodium scores in patients awaiting liver transplant: A cross-sectional study

**DOI:** 10.1177/00045632231196052

**Published:** 2023-08-20

**Authors:** Fatima Rodriguez-Alvarez, Paulina Moctezuma-Velázquez, Blanca Zuleyma Mota-Ayala, Paul Alonso Pamila-Tecuautzin, Ignacio García-Juárez, Carlos Moctezuma-Velázquez

**Affiliations:** 1 Gastroenterology Department, Instituto Nacional de Ciencias Médicas y Nutrición Salvador Zubirán, Mexico City, Mexico; 2 Surgery Department, 42559 Instituto Nacional de Ciencias Médicas y Nutrición Salvador Zubirán, Mexico City, Mexico; 3 Division of Gastroenterology (Liver Unit), 3158 University of Alberta, Edmonton, AB, Canada

**Keywords:** Hyponatremia, hypoalbuminemia, cirrhosis, model for end stage liver disease, transplant

## Abstract

**Introduction and aims:**

Sodium can be measured with direct or indirect methods; abnormal plasma total protein concentration can impact on sodium measured by indirect ion-selective electrodes (ISE). Serum sodium is an important item to determine the Model for End Stage Liver Disease Sodium (MELD-Na) score, commonly used for liver graft allocation. Patients with cirrhosis usually have hypoproteinemia. The aim of this study was to determine if there was a significant difference between the MELD-Na scores calculated based on the results of two different serum sodium ISE: indirect and direct.

**Methods:**

This was a retrospective study; we included 166 patients that underwent liver transplant assessment, and that had paired (*i.e.* same date and time) direct and indirect sodium determinations. We calculated the MELD-Na scores with both sodium determinations, and we compared them.

**Results:**

There was a significant difference between MELD-Na scores; the mean difference was 0.4±1.3. If MELD-Na score had been determined by the sodium measured by the direct ISE, 69 patients (42%) would have stayed in the same place on the waiting list, 67 patients (40%) would have moved up, and 30 patients (18%) would have moved down.

**Conclusions:**

There was a statistically significant difference between the MELD-Na scores calculated based on the two different sodium concentrations, which would theoretically result in changes in the order of the waiting list. This finding should prompt studies to assess if MELD-Na calculated based on direct methods has a better performance to predict clinically relevant outcomes.

## Introduction

There are two commonly used methods to determine sodium concentrations: the indirect ion-selective electrodes (ISE), which includes a pre-analytic dilution, and the direct ISE, as in blood gas analysers, where no dilution is needed. Most laboratories use the indirect ISE. When the indirect method is used, hyperlipidemia or increased protein concentrations may cause pseudohyponatremia, whereas decreased protein concentrations (*e.g.* hypoalbuminemia) may cause pseudonormonatremia. Paired comparisons between these two methods have found a mean difference of 2.1 mmol/L in sodium concentrations; importantly, as the plasma water content deviates from normal, this difference may also increase.^1^ Some studies have shown a higher discrepancy between these methods, for example, Dimesky et al. found a difference of 4 mmol/L in 25% and 8% of the specimens from intensive care unit (ICU) and other hospital areas, respectively, and a rate of pseudonormonatremia of 19%.^2^

The Model of End Stage Liver Disease (MELD) score, which takes into account creatinine, bilirubin, and INR, was originally intended to predict 3-month mortality in patients with cirrhosis undergoing TIPS (transjugular intrahepatic portosystemic shunt) procedure.^3^ Then, in 2002, the MELD score was adopted by the United Network for Organ Sharing in United States to allocate grafts in the liver transplant program.^4^ Later on, the equation was modified to include Na (*i.e.* MELD-Na), due to the fact that hyponatremia carries a poor prognosis in these patients. Patients with cirrhosis and hyponatremia have a 5% increased risk of mortality with every mmol reduction in serum sodium between 125 and 140 mmol/L.^5^ This equation was adopted by UNOS in 2016, and patients are usually considered for liver transplant when their MELD-Na is ≥ 15, which is associated with an estimated 3-month mortality of 6%.^5^ The higher the MELD-Na value, the higher the 3-month mortality, so that patients with MELD-Na of 20, 30, and 40, have a 20%, 53%, and 71% 90-day mortality, respectively. False values of serum sodium concentrations in patients with cirrhosis may modify their MELD-Na, their estimated mortality risk, and their place in the liver transplant waiting list. More recently, MELD-Na has been modified to include sex and albumin, but this equation (*i.e.* MELD 3.0) is still not in use.^6^

This work aimed to determine if there was a significant difference between the MELD-Na calculated with serum sodium measured by a direct ISE [MELD-Na(D)] and by an indirect ISE [MELD-Na(I)] in patients with cirrhosis. A secondary objective was to determine the correlation between the difference between MELD-Na(D) and MELD-Na(I) and serum total protein (TP) and albumin concentrations.

## Material and methods

### Study population

This was a retrospective study performed at a single referral centre. We included all consecutive patients with liver cirrhosis that received a liver graft between 2015 and 2019, and that had simultaneous (*i.e.* same time and date) determination of sodium in arterial blood gases (direct method) and peripheral venous blood chemistry (indirect method). In our centre, liver transplant assessment includes an extensive lab-work panel that includes these two determinations, in part to rule out hepatopulmonary syndrome. We included 166 patients. The Institutional Review Board approved this study (Ref. No. **GAS-3659-21-21-1**); as such, this research is compliant will all National regulations, institutional policies, and is in accordance the tenets of the World Medical Association Declaration of Helsinki. Informed consent was waived because of the observational nature of the study.

### Data collection

Each patient´s medical records were reviewed to gather clinical and demographic data, including sodium results from arterial blood gas analysis and from the blood chemistry. The arterial blood gas analysis was determined using the ABL835 FLEX (Radiometer Medical ApS, Denmark) between 2015 and 2018, and with the ABL90 FLEX (Radiometer Medical ApS, Denmark) during 2019; both analysers use direct ISE technique. The blood chemistry analysis was performed with the AU2700 analyser (Beckman Coulter, Brea, USA) via indirect ISE. We calculated the MELD-Na based on the results provided by the ABL835 FLEX/ABL90 FLEX [MELD-Na(D)] and by the AU2700 analyser [MELD-Na(I)], and we then calculated the difference [MELD-Na(D) − MELD-Na(I)] between them.

### Data analysis

Categorical variables were reported as frequencies and percentages. Continuous variables were described using mean ± standard deviation (SD). We used the paired sample t-test to compare values between blood chemistry and arterial blood gases. Correlation between the different variables was evaluated with the Pearson´s correlation coefficient. We calculated the limits of agreement for paired sodium determinations based on the method described by Bland and Altman.^7^ Linear regression was performed to study the association between the MELD-Na(D) − MELD-Na (I) difference and other variables. We conducted a sub-analysis in patients with MELD<21 because previous research has shown hyponatremia to be particularly relevant in terms of prognosis in this group of patients.^8^ We conducted subgroup analysis by TP concentration, with low TP defined as <60 g/L, because the difference between direct and indirect Na is predicted to be larger in these patients. We also conducted subgroup analysis by Child–Pugh class. The Child–Pugh is a score that takes into account 5 variables (*i.e.* albumin, prothrombin time or INR, bilirubin, ascites, and hepatic encephalopathy) to classify patients into 3 classes: Child A are compensated patients with well-preserved liver function, and Child B and C patients are patients with more liver dysfunction. Regarding sample size calculation, there are no studies addressing changes in MELD-Na according to the method being used for Na determination. Considering a power of 80%, variance of 9 in the MELD score, and an expected difference of at least one MELD point, we calculated a sample size of 144 patients.^9^ Statistical analysis was performed using STATA (StataCorp. Stata Statistical Software: Release 14), p<.05 was considered statistically significant.

## Results

### Baseline characteristics

We included 166 patients whose baseline characteristics are summarized in Table 1. Na(D) was lower than Na(I) (132.6 ±5.8 and 133.4 ± 5.6 mmol/L, p<.001). The mean difference was −0.75 ± 2.4 mmol/L and the 95% limits of agreement were −3.9 to 5.4. This difference was −1.16 ± 2.4 mmol/L in the group of patients with TP<60 g/L, and −0.59 + 2.4 mmol/L in the group of patients with TP ≥ 60 g/L.

**Table 1.** General Characteristics.

Male, n(%)	80 (48)
Age, years ± SD	49.9 ± 12.4
Aetiology, n (%)	
AIH/PBC/PSC	59 (36)
MAFLD/cryptogenic cirrhosis	37 (22)
Hepatitis C Virus	26 (16)
Hepatocellular carcinoma	23 (14)
Alcohol	20 (12)
Hepatitis B virus	1 (0.6)
Total bilirubin, umol/L ± SD	114.6 ± 126.6
Creatinine, umol/L ± SD	97.2 ± 70.7
INR, mean ± SD	1.5 ± 0.5
Albumin, g/L ± SD	29 ± 7.0
Serum total protein, g/L ± SD	67 ± 11
Aspartate aminotransferase, U/L ± SD	115.8 ± 185.0
Alanin aminotransferase, U/L ± SD	73.2 ± 114.3
Alkaline phosphatase, U/L ± SD	235.3 ± 234.5
Platelets, 10^9^ x L ± SD	88.3 ± 58.2
Na (I), mmol/L ± SD	133.4 ± 5.6
Na (D), mmol/L ± SD	132.6 ± 5.8
Child–Pugh A/B/C, n (%)	18 (11)/77 (46)/71 (43)
MELD, mean ± SD	17.8 ± 6.4

AIH: Autoimmune hepatitis; INR: International normalized ratio; MELD: Model for End Stage Liver Disease; Na (I): Sodium measured with indirect ion-selective electrode; Na (D): Sodium measured with direct ion-selective electrode; PBC: Primary biliary cholangitis; PSC: Primary sclerosing cholangitis; SD: Standard deviation.

### Comparison between MELD-Na (D) and MELD-Na(I)

**Figure 1.**
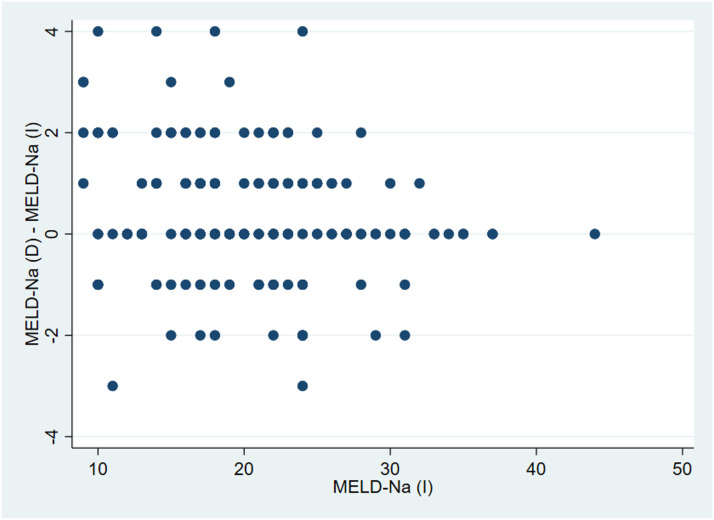
Distribution of the difference between MELD-Na(D) and MELD-Na(I) and MELD-Na (I).

There was a statistically significant difference between mean MELD-Na(D) and MELD-Na(I) (Table 2). The difference between MELD-Na(D) and MELD-Na(I) showed wide scatter for individual patients (Figure 1).

**Table 2.** Comparison and Correlation Between MELD-Na (D) and MELD-Na (I) by Child–Pugh Class, MELD, and Serum Total Protein.

	MELD-Na(D)	MELD-Na(I)	p	Correlation (MELD-Na)	Mean difference
Overall (n = 166)	21.0 ± 6.6	20.6 ± 6.8	<0.001	0.98, p < .001	0.4 ± 1.3
Child A (n = 18)	12.6 ± 3.8	11.8 ± 3.6	0.08	0.90, p < .001	0.7 ± 1.7
Child B (n = 77)	20.5 ± 6.2	20.1 ± 6.2	0.007	0.98, p < .001	0.4 ± 1.3
Child C (n = 71)	23.6 ± 5.6	23.2 ± 5.9	0.01	0.98, p < .001	0.4 ± 1.2
MELD<21 (n = 123)	18.3 ± 4.7	17.8 ± 4.8	<0.001	0.96, p < .001	0.5 ± 1.4
TP<60 g/L (n = 44)	24.5 ± 6.4	23.8 ± 6.6	<0.001	0.99 p < .001	0.7 ± 1.1
TP≥60 g/L (n = 122)	19.7 ± 6.2	19.4 ± 6.4	0.009	0.98 P < .001	0.3 ± 1.4

MELD: Model for End Stage Liver Disease; TP: Serum total protein.

**Figure 2.**
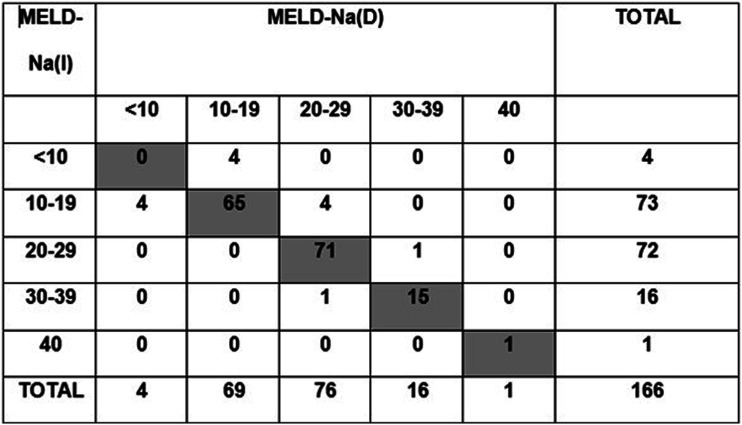
Distribution of MELD-Na(I) and MELD-Na(D) scores. Coloured cells demonstrate the patients who had the same score with both determinations.

Overall, if MELD-Na(D) had been used for organ allocation instead of the MELD-Na(I), 69 patients (42%) would have stayed in the same place, 67 patients (40%) would have moved up in the waiting list, and 30 patients (18%) would have moved down. In the group of patients with MELD<21, 57 (46%) would have moved up, compared to only 10 (23%) in patients with MELD≥21. In the group of patients with TP<60 g/L, 2 (4.5%) would have moved up and none down, whereas in the group of patients with TP ≥ 60 g/L, 7 (5.7%) would have moved up and 5 (4.1%) down (Figure 2 and Supplemental Figure 1).

### Association between MELD-Na(D)/MELD-Na(I) difference and other variables

**Figure 3.**
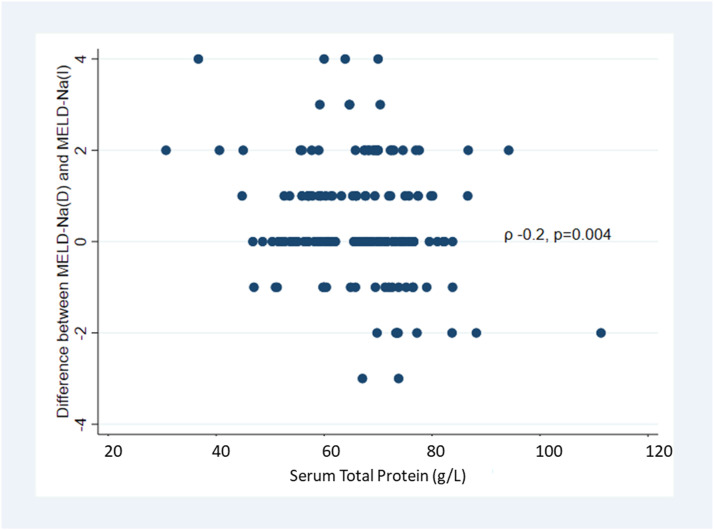
Scatter plot showing the correlation between the MELD-Na(D) − MELD-Na(I) difference and total serum protein concentration.

In linear regression analysis, TP concentration was associated with the difference between the MELD scores (β = −0.27 [95% CI: −0.45 to −0.09], p = .004), and there was a significant but very weak inverse correlation between these variables (ρ = −0.2, p = .004) (Figure 3). There was no association between MELD-Na difference and other variables of interest such as albumin [β = 0.06, (95% CI: −0.22 to 0.33), p =.7] or Child–Pugh score (β = −0.03, [95% CI: −0.13 to 0.07], p = .6).

## Discussion

Serum sodium can be determined with direct and indirect methods. Indirect methods can be influenced by TP concentrations, which are usually abnormal in patients with liver cirrhosis, as was recently underscored in an editorial.^10^ MELD-Na is currently the standard score used for liver transplant allocation and is usually based on the Na reported in the blood chemistry (*i.e.* indirect method). Therefore, the aim of this study was to compare the MELD-Na(D) with the MELD-Na(I), and to assess if the difference between them was associated with TP concentrations.

Although the mean difference between Na(D) and Na(I) was only −0.75 mmol/L in our study, the 95% limits of agreement suggest that the magnitude of this difference can be clinically relevant in individual patients, and this may become even more important when translated into changes in the MELD-Na score. For instance, in patients where the difference reached 2.3 mmol/L, their mortality risk could have been under or overestimated by up to 10%. Ray Kim et al. described that for every unit of decreased sodium concentration between 125 and 140 mmol/L the mortality risk increased by 5%; hence, pseudonormonatremia resulting from the use of indirect methods could underestimate mortality risk.^5^ Mirzazadeh et al. found a similar magnitude of mean difference between Na(D) and Na(I) of −0.57 mmol/L in an analysis of almost 20,000 paired blood gas and laboratory blood samples.^11^ In contrast, Story et al. found a larger mean difference of 2.1 mmol/L in 300 critically ill patients admitted to the ICU.^1^ There are 3 factors that may contribute to the smaller mean difference in Na in our study: first, their patients had lower albumin levels compared to ours; second, both sodium determinations were conducted in arterial blood, and in our case, it is arterial and venous; third, they used a different analyser, and the magnitude of this difference is analyser-dependent in the case of indirect ISE.^12^ It is worth mentioning that other factors such as dilution from heparin in syringes used to draw samples for blood gas analysis may contribute to differences between the methods.^13^

Although there was a strong correlation between MELD-Na(D) and MELD-Na(I), and the mean difference between them was only 0.4±1.3, we did find a statistically significant difference between the two scores. The consequence of this is that MELD-Na (I) may on average underestimate the risk as compared to MELD-Na (D). This finding may be relevant for liver allocation purposes, where a one-point change in the MELD-Na score could be a turning point for an organ recipient. For example, in our study, the mean increase in MELD points was significantly higher with the MELD-Na (D) when compared to the MELD-Na(I), so that 40% patients, would have moved up in the waiting list if the MELD-Na(D) had been used instead, and 5.4% would have been reclassified to a higher MELD tier. This is significant when compared to the 10.1% up-categorization shown with MELD 3.0.^6^ It is important to mention that according to the MELD-Na equation, the score only increases in presence of hyponatremia, so patients with pseudonormonatremia will not receive the net benefit of using the MELD-Na if using indirect methods to measure Na.^14^ The discrepancy between the two scores was not constant, in some cases, MELD-Na(D) was higher, but in some of them, it was lower or it had no change, which means there is no simple arithmetic way to transform MELD-Na(I) into MELD-Na(D).

As prior studies showed that it was primarily in patients with relatively low MELD scores (i.e. <21) that hyponatremia was associated with an increased risk of mortality (8), we performed a subgroup analysis in patients with MELD score <21, and in this subgroup, the mean difference reached 0.5±1.4 points. The difference between the MELD scores was the highest in well-compensated patients (*i.e.* Child A); however, it is in this specific group of patients where this difference would be less relevant as these patients are usually not at the top of the waiting list. On the other hand, in patients with MELD scores over 30, sodium concentrations are not determinant for mortality risk.^5^

TP concentrations were only weakly correlated with the difference between MELD-Na(D) and MELD-Na(I), and therefore, we think would not be useful as a correction factor in these patients, as some have stated before in other scenarios.^15^ Serum sodium deviations as high as 4 mmol/L have been described in samples of patients with TP concentrations lower than 40 g/L, but because the total protein concentration in our sample was on average slightly lower than normal, there was a tendency for the Na (I) to be only slightly higher than Na (D). Instead, we decided to split our data using the bottom of the TP reference range, 60 g/L,^15^ and as expected, the difference between MELD-Na(D) and MELD-Na(I) was greater in patients with low TP.

Our study does have some limitations. First, its retrospective and cross-sectional design, and the fact that all included patients had undergone a liver transplant, prevented us from evaluating the impact of our findings on clinical outcomes, such as prediction of mortality and/or decompensation. Second, even if samples were from the same time and date, one of them was arterial and the other venous, so it is possible that some of the divergences between results could be due to this fact. Third, most patients did not have same time and date lipid profiles, so we could not evaluate their association with Na concentrations. Finally, even if acute-on-chronic liver failure is not a common indication for liver transplant in our centre, and therefore, most transplant workups were performed on an outpatient basis, we cannot exclude the possibility that some patients may have been undergoing processes at the time of blood collection, which could further modify differences between the two methods. For example, drawing samples proximal to a catheter can result in a diluted blood sample, and albumin infusions can change the albumin concentration of a patient from one time point to another.

## Conclusions

In conclusion, there was a statistically significant difference between the MELD-Na(D) and the MELD-Na(I) scores. A prospective study should be conducted to compare the performance of MELD-Na(D) and MELD-Na(I) in predicting clinically relevant outcomes, which would be the only reason to prompt a change in the MELD-Na of this nature.

## Supplemental material

Supplemental material - Different serum sodium assay, different MELD-Na scores in patients awaiting liver transplant: A cross-sectional study Supplemental material for Different serum sodium assay, different MELD-Na scores in patients awaiting liver transplant: A cross-sectional study by Fatima Rodriguez-Alvarez, Paulina Moctezuma-Velázquez, Blanca Zuleyma Mota-Ayala, Paul Alonso Pamila-Tecuautzin, Ignacio García-Juárez, and Carlos Moctezuma-Velázquez in Annals of Clinical Biochemistry.

## Appendix

### Abbreviations

MELD-Na: Model for End-stage Liver Disease Sodium Score
ICU: intensive care unit
ISE: ion-selective electrode
TP: serum total protein

## References

[1] Story DA Morimatsu H Egi M , et al. The effect of albumin concentration on plasma sodium and chloride measurements in critically ill patients. Anesth Analg 2007; 104(4): 893–897. 17377102 10.1213/01.ane.0000258015.87381.61

[2] Dimeski G Morgan TJ Presneill JJ , et al. Disagreement between ion selective electrode direct and indirect sodium measurements: estimation of the problem in a tertiary referral hospital. J Crit Care 2012; 27(3): 326–416. 10.1016/j.jcrc.2011.11.003 22227082

[3] Malinchoc M Kamath PS Gordon FD , et al. A model to predict poor survival in patients undergoing transjugular intrahepatic portosystemic shunts. Hepatology 2000; 31(4): 864–871. 10733541 10.1053/he.2000.5852

[4] Kamath PS Wiesner RH Malinchoc M , et al. A model to predict survival in patients with end-stage liver disease. Hepatology 2001; 33(2): 464–470. 11172350 10.1053/jhep.2001.22172

[5] Kim WR Biggins SW Kremers WK , et al. Hyponatremia and mortality among patients on the liver-transplant waiting list. N Engl J Med 2008; 359(10): 1018–1026. 18768945 10.1056/NEJMoa0801209 PMC4374557

[6] Kim WR Mannalithara A Heimbach JK , et al. MELD 3.0: The Model for End-Stage Liver Disease Updated for the Modern Era. Gastroenterology 2021; 161(6): 1887–1895. 34481845 10.1053/j.gastro.2021.08.050 PMC8608337

[7] Bland JM Altman DG . Statistical methods for assessing agreement between two methods of clinical measurement. Lancet 1986; 1(8476): 307–310. 2868172

[8] Biggins SW Kim WR Terrault NA , et al. Evidence-based incorporation of serum sodium concentration into MELD. Gastroenterology 2006; 130(6): 1652–1660. 16697729 10.1053/j.gastro.2006.02.010

[9] Cengage Learning . BR Fundamentals of Biostatistics. 8th ed. Boston, MA: Cengage Learning, 2015, p. 888.

[10] Morton A . Measuring serum sodium in cirrhosis: regarding “hyponatremia in cirrhosis: an update”. Am J Gastroenterol 2021; 116(4): 835. 10.14309/ajg.0000000000001011 33982959

[11] Mirzazadeh M Morovat A James T , et al. Point-of-care testing of electrolytes and calcium using blood gas analysers: it is time we trusted the results. Emerg Med J 2016; 33(3): 181–186. 26396233 10.1136/emermed-2015-204669

[12] Tel-Karthaus N Salet GAM Jacobs LHJ , et al. Instrument dependent erroneous sodium measurements in hypoproteinemic critically ill patients are causing significant misclassification of dysnatremias. Clin Chem Lab Med 2019; 57(9): e222–e225. 30739096 10.1515/cclm-2018-0963

[13] Ustundağ Y Huysal K Ş O , et al. Interchangeability of sodium and potassium result values of arterial blood gas with laboratory analyzer: narrative review. Indian J Crit Care Med 2019; 23(1): 35–42. 31065207 10.5005/jp-journals-10071-23110 PMC6481262

[14] Freitas ACT Rampim AT Nunes CP , et al. Impact of meld sodium on liver transplantation waiting list. Arq Bras Cir Dig 2019; 32(3): e1460. 31826087 10.1590/0102-672020190001e1460 PMC6902892

[15] Katrangi W Baumann NA Nett RC , et al. Prevalence of Clinically Significant Differences in Sodium Measurements Due to Abnormal Protein Concentrations Using an Indirect Ion-Selective Electrode Method. J Appl Lab Med 2019; 4(3): 427–432. 31659081 10.1373/jalm.2018.028720

