# Supplementary material for: Different serum sodium assay, different model for end stage liver disease - sodium scores in patients awaiting liver transplant: A cross-sectional study
Source: Ann Clin Biochem. 2023 Aug 20;61(2):115–21. doi: 10.1177/00045632231196052 (PMC10938476; doi:10.1177/00045632231196052)

Supplementary Fig.1 Impact of using MELD-Na(D) compared to MELD-Na(I) depending on the original MELD score (*i.e.* lower or higher than 21)

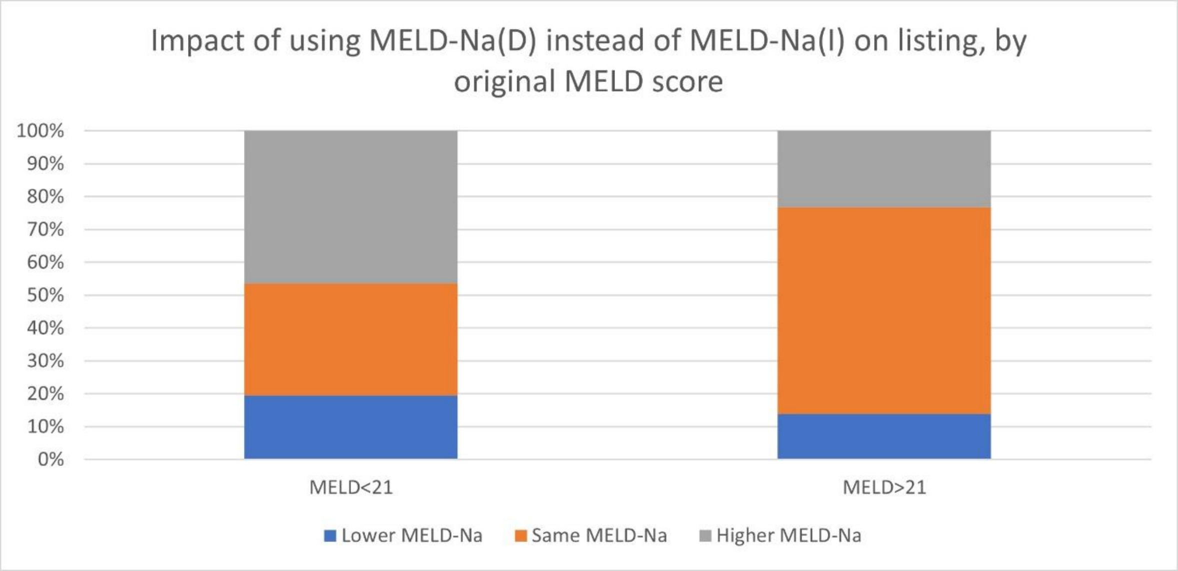

Supplement: Supplemental material - Different serum sodium assay, different MELD-Na scores in patients awaiting liver transplant: A cross-sectional study [file sj-pdf-1-acb-10.1177_00045632231196052.pdf]
